# Self-Esteem and Binge Eating among Adolescent Boys and Girls: The Role of Body Disinvestment

**DOI:** 10.3390/ijerph18147496

**Published:** 2021-07-14

**Authors:** Stefania Cella, Annarosa Cipriano, Cristina Aprea, Paolo Cotrufo

**Affiliations:** Observatory on Eating Disorders, Department of Psychology, University of Campania “Luigi Vanvitelli”, Viale Ellittico, 31-81100 Caserta, Italy; annarosa.cipriano@unicampania.it (A.C.); cristinaaprea97@gmail.com (C.A.); paolo.cotrufo@unicampania.it (P.C.)

**Keywords:** binge eating, adolescence, self-esteem, body disinvestment

## Abstract

Although low self-esteem and body disinvestment have been recognized as potential risk factors for disordered eating, no studies have explored how these factors may work together to predict binge eating in adolescents. Therefore, we hypothesized a path model for girls and boys separately to investigate whether the body’s investment dimensions (feelings towards the body, physical touch, body care, body protection) mediate the relationship between self-esteem and binge eating, and age moderate such relationships. Participants were 1046 Italian students aged between 11 and 19 years (472 girls, *M*_age_ = 14.17; 574 boys, *M*_age_ = 14.60) screened through self-report measures. Both models showed an acceptable fit (males: *χ*^2^_(22)_ = 30.441; RMSEA = 0.026; CFI = 0.99; TLI = 0.97; SRMR = 0.023; females: *χ*^2^_(22)_ = 34.723; RMSEA = 0.35; CFI = 0.98; TLI = 0.95; SRMR = 0.029). Negative body feelings and reduced body protection fully mediated the relationship between self-esteem and binge eating, regardless of gender. Our findings highlight the importance of interventions promoting body emotional investment to reduce adolescents’ vulnerability to binge eating.

## 1. Introduction

Binge eating refers to considerable consumption of food during a short period associated with feelings of loss of control (American Psychiatric Association, 2013). Researchers report that binge eating typically manifests during middle childhood and adolescence, with prevalence rates up to 45% [1]. A recent study reported that approximately 28% of children and adolescents aged 8–17 years have experienced objective and subjective binge eating episodes in the month prior to assessment [2]. Similarly, between 16.5% and 29.4% of adolescent boys and girls reported at least one binge eating day within the past month [3]. It has also been evidenced that females are more likely to report binge eating than males [4] and more severe indicators of loss of control during binge eating [5]. Engaging in binge eating during adolescence has been prospectively associated with persistent eating pathology in later adolescence and young adulthood [6]. Longitudinal studies have also demonstrated that binge eating is relatively stable over time [7] and can persist into adulthood [6]. Binge eating is strongly associated with overweight, obesity, symptoms of depression [8], and suicide attempts [9], and individuals with binge eating are more vulnerable to developing health problems [10] and poorer psychosocial outcomes [11]. Moreover, binge eating is the hallmark feature of bulimia nervosa (BN) and binge eating disorder (BED) [12], and episodes of binge eating are associated with a higher risk of developing full-syndrome eating disorders (EDs) [13]. 

An emerging body of evidence suggests that negative self-esteem and how adolescent experiences the body may confer vulnerability for binge eating in adolescence [14,15,16]. Given such variables’ interrelatedness, exploring the relationship between self-esteem, body disinvestment, and binge eating may be necessary to understand how the variables’ interplay can increase vulnerability to binge eating.

### 1.1. Body (Dis)Investment

Body image is a multidimensional concept, which encloses several dimensions [17]. According to Cash’s theorization [17], body image has two closely related yet conceptually distinct dimensions: evaluation and investment. Evaluation refers to the extent to which an individual is satisfied/unsatisfied with the body; investment refers to the cognitive, behavioral, and emotional aspects crucial for the body image experiences (e.g., self-preservation) in everyday life. Theoretical and empirical literature argues that how one perceives the body influences the process of emotional investment [18]. In this sense, adolescence is a critical stage for defining perceptions, thoughts, and feelings about own body and shaping body image due to the developmental challenges regarding bodily dimension [19]. Developmentally, differentiating bodily experience and somatic affect is a prerequisite to developing an adequate body self-awareness and self-integration [20]. Thus, youths are faced with integrating the pubertal body as part of body representation [21]. Relevant concerns over the body and its appearance typically emerge during early adolescence, as the attention of youth is channeled towards the developing physical body [22]. Puberty requires several changes and adjustments to manage the sudden biological instinctual urges and frightening sensations arising from the body [22]. A large body of research has shown that younger adolescents experience decreased self-esteem and increased negative body image [23,24]. Specifically, the pervasive sense of inadequacy in managing the increase of pubertal drives may disrupt the body’s relationship, leading to a detachment from the body—body disinvestment [25]—along with a less protective stance toward the body [26]. Such a body image disregard pattern stays stable or increases over adolescence and the transition into adulthood [23].

Specific body image disturbances constitute diagnostic criteria for anorexia nervosa (AN) and BN diagnoses [12]. Although disturbances in body image are not required for BED diagnosis [12], evidence suggests that individuals with BED show significantly greater impairment concerning body image than those without BED [27]. Given the strong association with disorder severity and adverse outcomes [28,29], body image disturbance has been suggested as a BED diagnostic specifier [30]. Therefore, it is not surprising that body image disturbances have been widely recognized as core clinical features in the etiology and maintenance of EDs [31,32] and disordered eating behaviors, such as binge eating. Specifically, in a longitudinal study exploring the risk factors for binge eating onset, Stice et al. [16] found that appearance overvaluation and body dissatisfaction predicted an increased risk for binge eating onset among adolescent girls. Similarly, individuals dissatisfied with their body image have greater odds of starting binge eating frequently [33]. In addition, preoccupations with weight and shape were found to be associated with binge eating concurrently, while weight and shape overvaluation was associated with increased odds of binge eating longitudinally, particularly in female participants [34]. Further confirming these results, an increased level of body satisfaction was found to predict binge eating cessation longitudinally [7]. 

### 1.2. Self-Esteem

Early models of EDs have argued that low self-esteem is involved in the etiology of eating pathologies [31]. Many of the subsequent theorizations have developed their models relying on the same constructs as the driving factor for binge eating pathology. For example, Stice et al. [16] have analyzed a set of potential binge eating risk factors concluding that low self-esteem predicts binge eating onset among adolescents. Cognitive-behavioral framework [14] and escape theory [35] have also emphasized the role of low self-esteem in precipitating binge eating. Empirical findings further confirm that self-esteem is a vulnerability factor for the development of EDs [36]. In a recent study, Cella et al. [37] have found that binge eating symptomatology was associated with a lower level of self-esteem. In this regard, a 5-years longitudinal study has demonstrated that improvement in self-esteem predicted greater odds of binge eating cessation [7]. However, the literature shows mixed results with regard to gender: low self-esteem seems to be a risk factor only for binge eating in girls [3], but these findings are not systematically replicated in other studies [7]. 

Poor self-esteem has also been recognized as a predisposing psychological factor for body image disturbance. For example, Paxton et al. [38] demonstrated that a lower level of self-esteem was a significant unique contributor in predicting negative body image. Lower self-esteem also negatively predicted body satisfaction among adolescent girls and boys [39]. Consistent with these findings, an educational school-based program focusing on improving self-esteem demonstrated positive effects on body image and long-term changes in body-related perceptions and attitudes [40]. 

### 1.3. The Present Study

Although several domains of body image, such as body dissatisfaction and weight and shape concerns, have been studied in binge eating research, highlighting their crucial role in developing and maintaining such dysfunctional behavior, to date no study has examined body emotional disinvestment in binge eating behaviors among adolescents. However, a recent study has demonstrated that such investment was negatively associated with ED symptoms. Specifically, negative feelings and attitudes towards the body, discomfort with physical touch and low levels of body protection significantly predicted a higher severity of disordered eating behaviors [41]. 

Moving from theoretical assumptions that body disinvestment constitutes a proximal factor in disordered eating behaviors due to the associated feelings of ineffectiveness and powerlessness [42], individuals who emotionally disinvest in this way may have a compromised relationship with their bodies [43] and be less able/willing to care for and protect the body, underestimating its importance [44]. As negative bodily experiences and perceptions may mobilize a sense of detachment from the body [26], and eating disorders constitute a threat to the body [45], examining such emotional disinvestment would fill an important gap in the binge eating literature, supporting the identification of specific risk factors for binge eating and helping to refine prevention and treatment practices.

**Figure 1:**
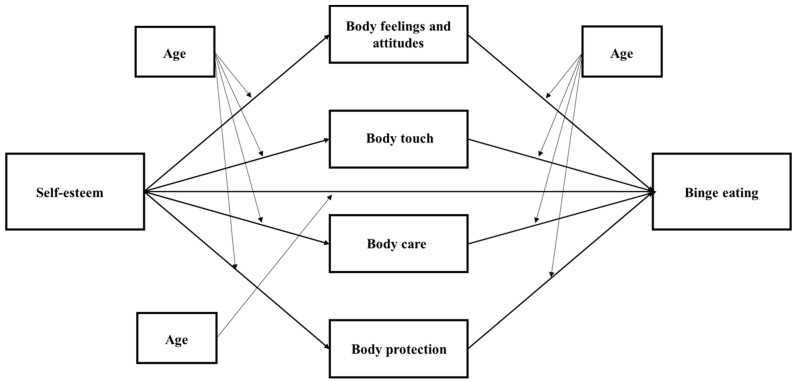
Model tested.

Thus, bearing these observations in mind, the present study aims to examine whether body emotional investment dimensions (feelings and attitudes towards the body, comfort with physical touch, body care, and body protection) mediates the relationship between self-esteem and binge eating behavior in a sample of adolescents. The model has been tested among adolescent boys and girls separately, as gender-specific risk factors are still unclear. Consistent with previous literature, it was hypothesized that self-esteem would directly affect binge eating, and such a relationship would be mediated by each facet of body emotional disinvestment. In addition, we investigated whether age moderates the model due to the relevance of such variables in the development process [46]. The model tested is shown in Figure 1. Body Mass Index (BMI) and socioeconomic status were introduced in the model as covariates, as the literature provides evidence regarding the association between binge eating, higher BMI and lower socioeconomic status [47,48].

## 2. Materials and Methods

### 2.1. Participants and Procedure

The sample comprised 1046 adolescents (*F* = 472, 45.1%) enrolled in middle and high schools located in Southern Italy. After school headmasters had agreed to participate in the study, parents’ informed consents and students’ oral assents were collected. The study took place during regular school hours, and research assistants were present at all times. Participants filled out a booklet of questionnaires as part of a larger study on eating attitudes. In addition, all participants were provided with information about local counselling resources and services. The study procedure was approved by the ethical committee of the institution involved and conducted in accordance with the guidelines of the Declaration of Helsinki [49].

### 2.2. Materials

#### 2.2.1. Self-Esteem

The Rosenberg Self-Esteem Scale (RSES) [50] was used to assess global self-worth. The RSES consists of 10 items (e.g., “*I feel I do not have much to be proud of*”), and responses are made on a 4-point Likert scale from 0 (“*Strongly agree*”) to 3 (“*Strongly disagree*”), with lower scores indicating lower self-esteem. The RSES has evidenced consistent reliability and validity [50]. In our sample, the internal consistency coefficient was 0.86. 

#### 2.2.2. Body Investment

The Body Investment Scale (BIS) [51] is a 24-item measure designed to assess emotional investment in the body. The BIS has a four-factor structure, with six items in each factor: feelings and attitudes towards the body (“*I feel comfortable with my body*”); body care (“*I believe that caring for my body will improve my well-being*”); body protection (“*It makes me feel good to do something dangerous*”); and comfort in physical touch (“*I like to touch people who are close to me*”). Respondents indicate the extent of agreement with each statement according to a 5-point Likert scale from 1 (“*I do not agree at all*”) to 5 (“*Strongly agree*”). Averaging the value of all items in each factor results in four separate sub-scores that are summed up to yield the overall total score. Lower scores indicate lower emotional investment in the body. The BIS showed sound psychometric properties [51]. Confirmatory factor analysis confirmed the four-factor solution in our sample (*χ*^2^_(223)_ = 765.02; RMSEA = 0.048; CFI = 0.92; TLI = 0.90; SRMR = 0.05). All factors were correlated with each other (*r* ranged from 0.101 to 0.501, *p* < 0.05). In the present study, the internal consistency for each subscale was: body feelings/attitudes *α* = 0.90; comfort in physical touch *α* = 0.69; body care *α* = 0.65; body protection *α* = 0.63; total score *α* = 0.79.

#### 2.2.3. Binge Eating

The Binge Eating Scale (BES) [52] is a 16-item self-report measure developed to assess the affective, cognitive (e.g., preoccupation with eating restriction, shame, guilt), and behavioural manifestations (e.g., eating fat, eating in secret, amount of food consumed) of binge eating. Each question has a group of numbered statements, and participants were asked to mark the statement in each group that best described how they felt or behaved. Through items 3, 9 and 12, the scale also assesses the individual’s perception of loss of control over one’s eating. Respondents rate each item on a 4-point Likert scale from 0 (no symptoms severity) to 3 (greater symptoms severity), with higher scores indicating severe binge eating problems. Although the BES was developed for use with obese adults, previous studies supported its use among community samples of adolescents and obese adolescents seeking treatment, showing high test-retest reliability (*r*  =  0.87) [53] and good internal consistency (Cronbach’s α = 0.79) [15]. In our study, the internal consistency was 0.87 for males and 0.86 for females.

#### 2.2.4. Body Mass Index

Each individual was weighed and measured in height.

### 2.3. Data Analysis

Descriptive statistics and correlational analyses were used to characterize the sample via SPSS v.26 (IBM, Armonk, NY, USA) [54]. The relationship between self-esteem, dimensions of body investment, and binge eating was evaluated separately for males and females, using the path model analysis in Mplus v7.4 (Muthén & Muthén, Los Angeles, CA, USA) [55]. Self-esteem was used as an exogenous variable, domains of body investment were introduced as mediators, and binge eating was treated as an endogenous variable (Figure 1). Age was introduced as a moderator of the model’s paths. The conditional indirect effect was estimated at low (−1SD), medium (mean), and high (+1SD) moderator levels in order to understand the nature of the moderated mediation effect. For the moderated mediation model, variables were centered on reducing multicollinearity. Background variables (socioeconomic status and BMI) were introduced in the model as covariates. Analyses were performed using the Maximum Likelihood (ML) estimation. The significance of the indirect effects was evaluated using the bootstrap procedure (5000 draws). A mediation effect is significant when the lower- and upper-level of the 95% bias-corrected confidence intervals (CI) do not include zero. Consistent with suggestions [56], a combination of both absolute and incremental indices was taken into account to evaluate the individual model fit: (a) the Chi-square statistic, which was used as a rough indicator of model fit due to its sensitivity to sample size [57], the Root Mean Square Error of Approximation (RMSEA) and the Standardized Root Mean Square Residual (SRMR) which should be less than 0.05 for an excellent fit [58], and the Comparative Fit Indices (CFI) and the Tucker-Lewis index (TLI) which should be higher than 0.90 for acceptable fit [56]. Each of these fit indices provides different information about the ability of the model to reproduce the input covariance matrix (RMSEA = fit adjusting for model parsimony; SRMR = absolute fit; CFI and TLI = fit relative to a null model) [56]. Considered together, these indices provide a more conservative and reliable evaluation of the fit of the model.

## 3. Results

### 3.1. Preliminary Analyses

Missingness was investigated through Little’s MCAR test [59]. A non-significant test statistic (*χ*^2^_(42)_ =  48.225; *p =* 0.23) indicated that the data were missing completely at random and not biased by missingness. Thus, the Expectation Maximization (EM) was used to impute missing data [60]. The analysis of skewness and kurtosis coefficients indicated that there was no severe violation of univariate and multivariate normality. Correlational analyses showed that all main variables were significantly correlated in the expected direction. Mean, standard deviations, inter-correlation, and alpha coefficients are reported in Table 1. 

### 3.2. Descriptive

Participants were 1046 students aged between 11 and 19 years (*M_age_* = 14.40, *SD* = 1.50). Of the sample, 574 (54.9%) were male (*M_age_* = 14.60, *SD* = 1.50), and 472 (45.1%) were female (*M_age_* = 14.17, *SD* = 1.47). About 60% (*n* = 591) fell into the lower- to upper-middle socioeconomic class, with no differences between socioeconomic groups (*χ*^2^_(1)_ = 3.333; *p* = 0.068). The majority (98.36%) reported being of Italian nationality. The average BMI was 22.07 (*SD* = 3.68) and no differences were found between gender (*t*_(1044)_ = 821; *p* = 0.412—Males: *M* = 22.16, *SD* = 3.73; Females: *M* = 21.97; *SD* = 3.62).

### 3.3. Path Analysis Model 

**Figure 2:**
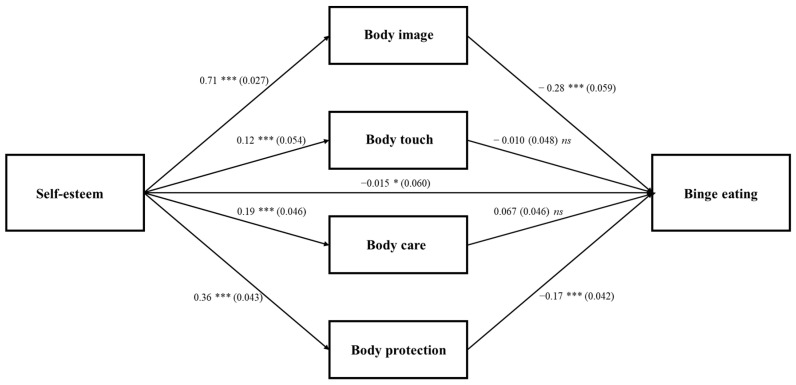
Females model: Standardized path analysis coefficients and standard errors * *p* < 0.05, *** *p* < 0.001, *ns*—not significant.

Both models provided a good fit to the data (males: *χ*^2^_(22)_ = 30.441; RMSEA = 0.026; CFI = 0.99; TLI = 0.97; SRMR = 0.023; females: *χ*^2^_(22)_ = 34.723; RMSEA = 0.35; CFI = 0.98; TLI = 0.95; SRMR = 0.029). All the direct paths were statistically significant among females, except for the path from body touch (*β* = −0.010, *p* = 0.83) and body care (*β* = 0.067, *p* = 0.15) to binge eating. (Figure 2). The direct effect of self-esteem on binge eating was statistically significant (*β* = −395, *p* < 0.000), but the path reduced in absolute size when the mediators were introduced (*β* = −0.153, *p* = 0.010). Thus, results showed that the indirect path from self-esteem to binge eating was partially mediated by body image feelings (*β* = −0.202, 95% CI [−0.276, −0.130]; *p* < 0.001) and body protection (*β* = −0.064, 95% CI [−0.096, −0.037]; *p* < 0.001). The model accounted for 27% of the variance in binge eating. Inspection of the conditional indirect effects for each level of the moderator revealed no moderation effect. Regarding covariates, BMI was statistically significant in predicting body image (*β* = −0.173, *p* < 0.000) and binge eating (*β* = 0.089, *p* = 0.016). 

**Figure 3:**
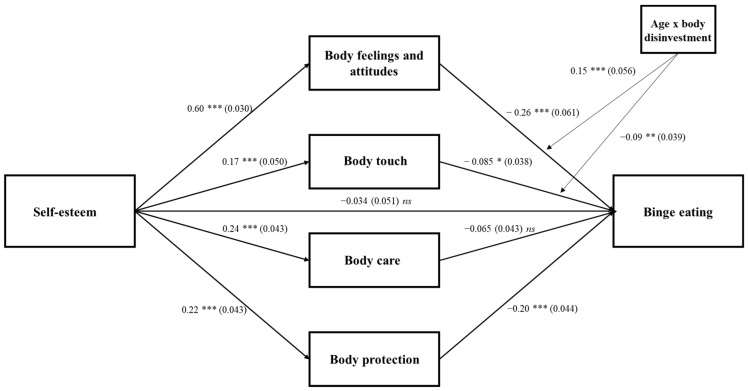
Males model: Standardized path analysis coefficients and standard errors * *p* < 0.05, ** *p* < 0.01, *** *p* < 0.001, *ns*—not significant.

All the direct paths were statistically significant among males, except for the path from body care (*β* = −0.065, *p* = 0.13) to binge eating (Figure 3). The direct effect of self-esteem on binge eating was statistically significant (*β* = −0.272, *p* < 0.000), but it became no longer significant after mediators had been controlled (*β* = −0.034, *p* = 0.50). Body image feelings (*β* = −0.158, 95% CI [−0.225, −0.096]; *p* < 0.001) and body protection (*β* = −0.044, 95% CI [−0.072, −0.025]; *p* = 0.001) significantly and fully mediated the path from self-esteem to binge eating. The model accounted for 26% of the variance in binge eating. Results showed a significant interaction between body image (*β* = −0.15, *p* = 0.005), body touch (*β* = −0.09, *p* = 0.010) and age in predicting binge eating. Inspection of the conditional indirect effects revealed that the index of second-stage moderated mediation from self-esteem to binge eating through body image feelings was significant for older adolescents (*β* = −0.67, 95% CI [−1.12, −0.40]; *p* = 0.001). Regarding covariates, BMI was statistically significant in predicting self-esteem (*β* = −0.093, *p* = 0.007) and body image (*β* = −0.220, *p* < 0.000). More details are reported in the Supplementary Materials (Table S1).

## 4. Discussion

The main aim of the present study was to examine, among male and female adolescents, whether the relationship between self-esteem and binge eating is mediated through body (dis)investment dimensions (i.e., body image feelings, body touch, body care, and body protection) and whether age acts as a moderator of the model’s path.

Contrary to our expectations, the path analysis model among males indicated that self-esteem did not directly affect binge eating. However, our findings are in line with previous literature suggesting that the pathway from self-esteem to binge eating is mediated by body image concerns [14]. However, the data also corroborate previous studies suggesting that negative feelings about the body and lower levels of self-protection are associated with disordered eating behaviors [41]. More specifically, the current study provides the first empirical evidence that body disinvestment acts as a mediator in the relationship between self-esteem and binge eating among both males and females. Results showed that the absence of bodily self-protection and negative feelings and attitudes towards the body accounted for more than 25% of the variance in binge eating, regardless of gender.

The dual pathway from self-esteem to binge eating, through negative body attitudes and feelings and reduced body protection, highlights that a pervasive negative self-evaluation may be detrimental for developing a functional body image due to emotional disinvestment. It could be argued that the ineffectiveness of managing pubertal drives and the trouble in integrating infant fantasies in the sexually mature body may disrupt the body’s relationship, leading to a detachment from it [25,61]. In turn, according to Orbach’s theorization [51], a distorted experience of the body and negative attitudes towards the body interfere with the self-preservation processes, facilitating self-destructive behaviors, such as suicide attempts, self-injurious behaviors, and disordered eating behaviors. In this sense, engaging in disordered eating behaviors, such as binge eating, may reflect a sense of detachment and deference towards the body (i.e., low protection and negative body attitudes). 

These results are also in line with the transdiagnostic cognitive-behavioral model of eating disorders [14]. According to such theory, the core psychopathology of eating disorders is a dysfunctional system for evaluating self-worth based on eating, shape and weight. This kind of self-worth evaluation is maintained by many different factors, such as low core self-esteem and emotional intolerance, and can explain other clinical features associated with eating disorders, such as body avoidance. Thus, body disinvestment could be a mechanism to deny the body as a source of poor self-worth evaluation.

The current model did not support the direct path from low care for the body to binge eating. It might be possible that items related to body care are less representative of body image experience in individuals engaging in disordered eating, as they are a step away from taking care of their body, and such a non-significant path may be related to denial of bodily existence. Similarly, although less comfort with body touch was correlated to binge eating behaviors, it failed to predict binge eating among females. 

From a theoretical perspective, such results seem to be in line also with emotional disinvestment in the body. As during adolescence, the body is a crucial object, the extraordinary demands of the pubescent body may posit limits to embracing a fuller sense of self and lead to developing a way of coping using the body [62]. It might be possible that eating disordered behaviors constitute an attempt to mortify the body in its appetites, denying the bodily changes [63]. As such, in the struggle for control over drives and urges, individuals with disordered eating established a sense of effectiveness [64,65].

The importance of controlling complex and negative feelings has also been supported by the negative affect pathway from Stice’s dual-pathway model [66], which proposes that binge eating may affect some individuals’ regulation functions.

As empirical research exploring such suggestions is still scarce, future studies would benefit from examining this research area, leading to refinement of current perspectives and more targeted prevention and intervention programs. 

Indices of moderated mediation for second-stage moderation suggest that self-esteem’s indirect effect on binge eating through body image feelings is significantly stronger among older boys. Thus, this result confirms that body disregard increases over time among males [23]. A potential explanation is that pubertal body changes may pull girls farther from the thin ideal body shape and, on the other hand, bring adolescent boys closer to the cultural ideal of a large, muscular man [67].

Our results expand the literature on binge eating, examining the relationship between self-esteem, body disinvestment, and binge eating, suggesting that, regardless of gender, body disinvestment may serve as a gender-shared mechanism for negative self-esteem, contributing to binge eating. 

Notwithstanding, important limitations need to be taken into account. The cross-sectional and correlational nature of our data limits any causal conclusions. Future longitudinal studies are required to ascertain the relationship’s temporal direction and support the mediational model described above. Self-report measures employed for data collection may result in bias inherent to this method, such as reporting bias and poor insight. Although BIS subscales demonstrated low internal consistency (α around 0.60), except for body image feelings, analyses confirmed the factor structure (four-factors) of the scale in our sample. However, conclusions must be interpreted with caution, pending replication. Moreover, the current study included a community sample of Italian adolescents, limiting the generalizability to other samples. Future research should cross-validate the mediational model among clinical samples of EDs individuals. 

## 5. Conclusions

Pathological manifestations of adolescence are expressed through the body, engaging in bodily-focused behaviors such as eating disorders [68]. As such, the present study provides promising evidence supporting the crucial role of body disinvestment as a mechanism through which low self-esteem influences binge eating engagement among adolescents. To date, the majority of studies have focused on the satisfaction/dissatisfaction component of body image, underestimating the importance of body investment and limiting a deeper conceptualization of binge eating behaviors. These results suggest that body investment, alongside body dissatisfaction, may be a critical factor in the development of binge eating among male and female adolescents and should be considered in the etiological models of binge eating. Our findings also have some practical implications. Results indicate that working on body investment may be a helpful avenue in facing binge eating. A clearer picture of which body image dimensions are related to binge eating would help clinicians in identifying adolescents at heightened risk for binge eating. In this sense, practitioners should promote prevention and intervention programs targeting acceptance and positive investment in the body to reduce vulnerability to binge eating behaviors among the young.

## Figures and Tables

**Table 1:** Means, standard deviations, and correlation coefficients of study variables.

**Males**	***M***	***SD***	**2.**	**3.**	**4.**	**5.**	**6.**	**α**
**Variables**								
1. Binge eating	6.10	6.98	−291 **	−421 **	−0.174 **	−0.185 **	−0.319 **	0.87
2. Self-esteem	20.99	5.49	-	0.621 **	0.168 **	0.231 **	0.230 **	0.84
3. Body image feelings	3.95	0.83		-	0.158 **	0.215 **	0.312 **	0.86
4. Body touch	3.47	0.63			-	0.345 **	0.166 **	0.63
5. Body care	3.60	0.62				-	0.186 **	0.63
6. Body protection	3.74	0.67					-	0.63
**Females**	***M***	***SD***	**2.**	**3.**	**4.**	**5.**	**6.**	**α**
**Variables**								
1. Binge eating	7.39	6.72	−404 **	−453 **	−0.036	−0.026	−0.305 **	0.85
2. Self-esteem	17.78	5.88	-	0.723 **	0.132 **	0.198 **	0.364 **	0.86
3. Body image feelings	3.58	1.01		-	0.053	0.072	0.306 **	0.91
4. Body touch	3.39	0.72			-	0.272 **	0.106 **	0.76
5. Body care	3.94	0.55				-	0.203 **	0.63
6. Body protection	3.90	0.65					-	0.62

** *p* < 0.01.

## Data Availability

The data that support the findings of this study are available from the corresponding author, S.C., upon reasonable request.

## Supplementary Materials

The following are available online at https://www.mdpi.com/article/10.3390/ijerph18147496/s1, Table S1: Standardized path coefficients for the moderated mediation models tested.
