# Supplementary material for: Self-Esteem and Binge Eating among Adolescent Boys and Girls: The Role of Body Disinvestment
Source: Int J Environ Res Public Health. 2021 Jul 14;18(14):7496. doi: 10.3390/ijerph18147496 (PMC8304970; doi:10.3390/ijerph18147496)
Supplement: Supplementary file 1 [file ijerph-18-07496-s001.zip › ijerph-1275319-supplementary.pdf]

**Article title**: Self-esteem and binge eating among adolescent boys and girls: the role of body disinvestment

**Journal name**: International Journal of Environmental Research and Public Health

**Authors names**: Stefania Cella, Annarosa Cipriano, Cristina Aprea, and Paolo Cotrufo

**Affiliation and e-mail address of the corresponding author**: Observatory on Eating Disorders, Department of Psychology, University of Campania "Luigi Vanvitelli", Viale Ellittico, 31 – 81100 – Caserta, Italy; stefania.cella@unicampania.it

**Supplementary Material**

**Table S1**: Standardized path coefficients for the moderated mediation models tested

| Males model | | | |
|---|---|---|---|
| **Variables** | ***β*** | **Standard error** | ***p*** |
| **Covariate: BMI** | | | |
| Self-esteem | **−0.093** | **0.034** | **0.007** |
| Body image | **−0.220** | **0.042** | **0.000** |
| Body touch | −0.009 | 0.032 | 0.772 |
| Body care | −0.044 | 0.027 | 0.097 |
| Body protection | −0.027 | 0.039 | 0.497 |
| Binge eating | 0.101 | 0.075 | 0.178 |
| **Covariate: Socioeconomic status** | | | |
| Self-esteem | 0.030 | 0.058 | 0.609 |
| Body image | −0.030 | 0.035 | 0.384 |
| Body touch | 0.004 | 0.039 | 0.910 |
| Body care | 0.016 | 0.031 | 0.613 |
| Body protection | −0.031 | 0.016 | 0.053 |
| Binge eating | −0.064 | 0.055 | 0.243 |
| **Outcome: Body Image** | | | |
| Self-esteem | **0.601** | **0.030** | **0.000** |
| Age | 0.012 | 0.031 | 0.683 |
| Age x Self-esteem | 0.028 | 0.040 | 0.487 |
| **Outcome: Body Touch** | | | |
| Self-esteem | **0.172** | **0.050** | **0.001** |
| Age | **0.094** | **0.043** | **0.027** |
| Age x Self-esteem | 0.094 | 0.052 | 0.072 |
| **Outcome: Body Care** | | | |
| Self-esteem | **0.245** | **0.043** | **0.000** |
| Age | **0.171** | **0.041** | **0.000** |
| Age x Self-esteem | 0.093 | 0.048 | 0.055 |
| **Outcome: Body Protection** | | | |
| Self-esteem | **0.222** | **0.047** | **0.000** |
| Age | **−0.111** | **0.046** | **0.016** |

| | | | | |
|---|---|---|---|---|
| Age x Self-esteem | −0.011 | 0.054 | 0.836 | |
| **Outcome: Binge eating** | | | | |
| Self-esteem | −0.034 | 0.051 | 0.501 | |
| Age | −0.020 | 0.039 | 0.617 | |
| Age x Self-esteem | −0.076 | 0.051 | 0.138 | |
| Body image | **−0.264** | **0.061** | **0.000** | |
| Age x Body image | **0.157** | **0.056** | **0.005** | |
| Body touch | **−0.085** | **0.038** | **0.025** | |
| Age x Body touch | **−0.099** | **0.039** | **0.011** | |
| Body care | −0.065 | 0.043 | 0.131 | |
| Age x Body care | −0.005 | 0.041 | 0.902 | |
| Body protection | **−0.201** | **0.043** | **0.000** | |
| Age x Body protection | −0.017 | 0.044 | 0.691 | |

| **Indirect Effect** | ***β*** | **Standard error** | ***p*** | **95% CI (Low- Upper)** |
|---|---|---|---|---|
| Self-esteem →Body image → Binge eating | **−0.158** | **0.039** | **0.000** | **−0.225; −0.096** |
| Self-esteem →Body touch → Binge eating | −0.015 | 0.008 | 0.059 | −0.031; 0.000 |
| Self-esteem → Body care → Binge eating | −0.016 | 0.011 | 0.149 | −0.036; 0.001 |
| Self-esteem →Body protection → Binge eating | **−0.044** | **0.014** | **0.001** | **−0.072; −0.025** |

| **Females model** | | | | |
|---|---|---|---|---|
| **Variables** | ***β*** | **Standard error** | ***p*** | |
| **Covariate: BMI** | | | | |
| Self-esteem | −0.057 | 0.044 | 0.191 | |
| Body image | **−0.173** | **0.031** | **0.000** | |
| Body touch | 0.048 | 0.043 | 0.257 | |
| Body care | 0.014 | 0.040 | 0.729 | |
| Body protection | −0.007 | 0.038 | 0.855 | |
| Binge eating | **0.089** | **0.037** | **0.016** | |
| **Covariate: Socioeconomic status** | | | | |
| Self-esteem | −0.040 | 0.046 | 0.382 | |
| Body image | 0.007 | 0.032 | 0.832 | |
| Body touch | 0.032 | 0.045 | 0.473 | |
| Body care | −0.032 | 0.043 | 0.458 | |
| Body protection | −0.032 | 0.042 | 0.453 | |
| Binge eating | 0.015 | 0.042 | 0.715 | |
| **Outcome: Body Image** | | | | |
| Self-esteem | **0.713** | **0.027** | **0.000** | |
| Age | −0.004 | 0.031 | 0.890 | |
| Age x Self-esteem | −0.038 | 0.033 | 0.255 | |
| **Outcome: Body Touch** | | | | |

| | | | |
|---|---|---|---|
| Self-esteem | **0.127** | **0.054** | **0.019** |
| Age | 0.091 | 0.051 | 0.074 |
| Age x Self-esteem | −0.036 | 0.058 | 0.534 |
| **Outcome: Body Care** | | | |
| Self-esteem | **0.194** | **0.046** | **0.000** |
| Age | **0.199** | **0.044** | **0.000** |
| Age x Self-esteem | 0.085 | 0.047 | 0.070 |
| **Outcome: Body Protection** | | | |
| Self-esteem | **0.361** | **0.043** | **0.000** |
| Age | −0.032 | 0.046 | 0.487 |
| Age x Self-esteem | −0.046 | 0.052 | 0.385 |
| **Outcome: Binge eating** | | | |
| Self-esteem | **−0.153** | **0.060** | **0.011** |
| Age | 0.021 | 0.042 | 0.610 |
| Age x Self-esteem | 0.026 | 0.054 | 0.626 |
| Body image | **−0.281** | **0.059** | **0.000** |
| Age x Body image | −0.026 | 0.054 | 0.624 |
| Body touch | −0.010 | 0.048 | 0.837 |
| Age x Body touch | −0.011 | 0.041 | 0.791 |
| Body care | 0.067 | 0.046 | 0.151 |
| Age x Body care | 0.087 | 0.045 | 0.051 |
| Body protection | **−0.175** | **0.042** | **0.000** |
| Age x Body protection | −0.050 | 0.037 | 0.178 |

| **Indirect Effect** | ***β*** | **Standard error** | ***p*** | **95% CI (Low-Upper)** |
|---|---|---|---|---|
| Self-esteem →Body image → Binge eating | **−0.202** | **0.044** | **0.000** | **−0.276; −0.130** |
| Self-esteem →Body touch → Binge eating | −0.001 | 0.007 | 0.853 | −0.014; 0.008 |
| Self-esteem →Body care → Binge eating | 0.013 | 0.009 | 0.166 | 0.000; 0.030 |
| Self-esteem →Body protection → Binge eating | **−0.064** | **0.018** | **0.001** | **−0.096; −0.037** |
